# Supplementary material for: Is studying medicine good for your health? Long-term health outcomes of a cohort of clinical medicine graduates in England and Wales in the ONS Longitudinal Study
Source: BMJ Open. 2021 Mar 18;11(3):e041224. doi: 10.1136/bmjopen-2020-041224 (PMC7978287; doi:10.1136/bmjopen-2020-041224)
Supplement: Author's manuscript [file bmjopen-2020-041224.draft_revisions.pdf]

BMJ Open

BMJ Open is committed to open peer review. As part of this commitment we make the peer review history of every article we publish publicly available.

When an article is published we post the peer reviewers' comments and the authors' responses online. We also post the versions of the paper that were used during peer review. These are the versions that the peer review comments apply to.

The versions of the paper that follow are the versions that were submitted during the peer review process. They are not the versions of record or the final published versions. They should not be cited or distributed as the published version of this manuscript.

BMJ Open is an open access journal and the full, final, typeset and author-corrected version of record of the manuscript is available on our site with no access controls, subscription charges or pay-per-view fees (http://bmjopen.bmj.com).

If you have any questions on BMJ Open's open peer review process please email info.bmjopen@bmj.com

BMJ Open

Is studying medicine good for your health? Long term health outcomes of a cohort of clinical medicine graduates in England and Wales in the ONS Longitudinal Study.

Journal:	*BMJ Open*
Manuscript ID	bmjopen-2020-041224
Article Type:	Research
Date Submitted by the Author:	04-Jun-2020

Complete List of Authors:	Shelton, Nicola; UCL, Department of Epidemiology and Public Health<br>Duke-Williams, Oliver; UCL, Department of Information Studies<br>van de Erve, Laura; Institute for Fiscal Studies<br>Britton, Jack; Institute for Fiscal Studies<br>Xun, Wei; University College London Institute of Epidemiology and Health Care

Keywords:	MEDICAL EDUCATION & TRAINING, EPIDEMIOLOGY, PUBLIC HEALTH, EDUCATION & TRAINING (see Medical Education & Training)

SCHOLARONE™
Manuscripts

I, the Submitting Author has the right to grant and does grant on behalf of all authors of the Work (as defined in the below author licence), an exclusive licence and/or a non-exclusive licence for contributions from authors who are: i) UK Crown employees; ii) where BMJ has agreed a CC-BY licence shall apply, and/or iii) in accordance with the terms applicable for US Federal Government officers or employees acting as part of their official duties; on a worldwide, perpetual, irrevocable, royalty-free basis to BMJ Publishing Group Ltd ("BMJ") its licensees and where the relevant Journal is co-owned by BMJ to the co-owners of the Journal, to publish the Work in this journal and any other BMJ products and to exploit all rights, as set out in our licence.

The Submitting Author accepts and understands that any supply made under these terms is made by BMJ to the Submitting Author unless you are acting as an employee on behalf of your employer or a postgraduate student of an affiliated institution which is paying any applicable article publishing charge ("APC") for Open Access articles. Where the Submitting Author wishes to make the Work available on an Open Access basis (and intends to pay the relevant APC), the terms of reuse of such Open Access shall be governed by a Creative Commons licence – details of these licences and which Creative Commons licence will apply to this Work are set out in our licence referred to above.

Other than as permitted in any relevant BMJ Author's Self Archiving Policies, I confirm this Work has not been accepted for publication elsewhere, is not being considered for publication elsewhere and does not duplicate material already published. I confirm all authors consent to publication of this Work and authorise the granting of this licence.

For peer review only

Is studying medicine good for your health? Long term health outcomes of a cohort of clinical medicine graduates in England and Wales in the ONS Longitudinal Study

Nicola Shelton[1]* Oliver Duke-Williams[2] Laura van der Erve[3] Jack Britton[3] Wei Xun[1]

1 Centre for Longitudinal Study Information and User Support (CeLSIUS), Department of Epidemiology and Public Health, UCL, London, UK.

2 Department of Information Studies, UCL, London, UK.

3. The Institute for Fiscal Studies, London, UK.

*Corresponding Author: Centre for Longitudinal Study Information and User Support (CeLSIUS), Department of Epidemiology and Public Health, UCL, 1-19 Torrington Place, London WC1E 6BT, UK. Email: n.shelton@ucl.ac.uk Tel +442076795648

Word count, excluding title page, abstract, strengths and limitations and references, figures and tables 1636

Contributorship statement: NS and ODW devised the research idea and NS and ODW and WX collated the data and completed the analysis. LVDE and JB co-wrote the literature review and discussion and provided comment on the full draft text.

For peer review only

For peer review only

Abstract

Objective: to quantify the protective effect on health associated with study of a clinical medicine degree.

Design: Prospective population based cohort data collected at census and linked over time study: cohort born before 1975 and survived to 2011, Subgroup analysis on those who reported having a degree at 1991 census.

Setting: England and Wales population based including institutions.

Participants: 131,433 men 148,704 women. 13,391 men with degrees 8,143 women with degrees.

Main outcome measure: self-reported general health in 2011 based on logistic regression analysis.

Results: Male graduates had 86% higher odds of having good health than non-graduates after adjustment for age and socio-economic position (confidence intervals 1.77-1.97). Female graduates had 72% higher odds of having good health than non-graduates after adjustment for age and socio-economic position (confidence intervals 1.61-1.84).

Male clinical medicine graduates had 32% lower odds of having poor health than male humanities graduates after adjustment for age and socio-economic position (confidence intervals 0.52--0.90). Male social sciences graduates also had higher odds of having good health than male humanities graduates after adjustment for age and socio-economic position, but life sciences and physical science graduates did not. There were no significant differences by degree subject for women.

Conclusions: Male graduates in clinical medicine have lower odds of poor self-reported health. Knowledge of medicine may confer a health advantage for men above that of other degrees.

Strengths and Limitations of this Study

Large sample size (total N > 1 000 000). This is by far the largest nationally representative longitudinal dataset in the UK.

Length of follow-up available (40 years, 1971–2011 for main census data) with life events for ONS Longitudinal Study members available until about 2 years before the current year of analysis.

The high tracing rates contribute to the high linkage rate of LS members from census to census (88% 2001 to 2011).

No behavioural data.

Census is every 10 years, so updates are limited.

Introduction

Several studies have shown the health benefits of education and the gradient in this. Education to degree level confers a greater health advantage.

Freedman and Martin[1] found that education level accounted for declines in functional limitations among older Americans from 1984 to 1993, and high school graduate education was the most important in accounting for recent trends of the eight demographic and socioeconomic variables they considered.

Elo and Preston[2] found proportionate reductions in mortality for each one-year increase in schooling in the United States at ages 35-54 comparable to those estimated for a number of European countries by Valkonen[3]. The main difference they found between the United States and Europe was that in the U.S. mortality reduction with years of schooling was quite similar for both men and women, while in a number of European countries male mortality was more greatly reduced than female mortality with educational attainment.

Walseman et al[4] had explored if later life qualifications benefited health. Among respondents with no degree, a high school diploma, or a post–high school certificate at 25 years of age, attaining at least a bachelor's degree by midlife was associated with fewer depressive symptoms and better self-rated health at midlife compared with respondents who did not attain a higher degree by midlife.

Rogers et al[5] showed that educational degrees were associated with reduced mortality risk in three cohorts of U.S adults aged 25 and above in 1997–2002 though they showed more marked gender differences, with associations not significant in older women and were weaker in women than in men. Among males in all cohort groups, there were gradients by educational degree level in the risk of death. The overall educational degree gradient was evident in all cohorts of women, although the mortality advantages for those with postsecondary degrees are generally not as pronounced among women as among men.

More recently Buckles[6] found that college (university) education among white men born between 1942 and 1953 in the US was associated with lower mortality and higher earning, but also with higher levels of health insurance offering a pathway to better health outcomes in the US but not in the UK, but also greater reductions in smoking and higher levels of physical activity.

We highlight the role of education for several reasons. First, education is strongly associated with many health- related behaviours over the life cycle, which frequently are not measured directly in nationally representative surveys and administrative data. Further, unlike some other measures of socioeconomic status such as occupation and income, educational qualification data are straightforward to report, and are generally fixed for each individual relatively early in life. In addition, Higher Education participation in UK increased from 3.4% in 1950, to 8.4% in 1970, to 19.3% in 1990.[7]

There has also been considerable work looking at the earnings returns to different degree subjects,[8-9] and also work looking at the wider returns to attending higher education, including the health outcomes.[10-11] However, there has been very little investigation in the health returns by different degree subjects. The UK government is increasingly focussed on the returns to different degrees as government subsidies of different subject areas has increased significantly;[12] understanding these wider returns is therefore highly important.

For peer review only

The research looks at the health outcomes of adults by which degree they studied. Self-rated health correlates strongly with clinical assessments of morbidity and subsequent mortality. The hypothesis was that clinical medicine would confer health advantage. Graduates in humanities have lower salaries and lower employment rates in the UK than graduates in medicine and science (ONS, 2013)[13] yet there are no studies of how this related to health outcomes. This is the first study to consider the health benefits of studying different degree subjects.

We hypothesise that clinical medicine graduates will have lower odds of poor self-reported health.

Methods

The ONS Longitudinal Study (LS) comprises people born on one of four selected dates of birth and so makes up about 1% of the total population in England and Wales – that data is linked for five successive censuses starting at 1971; new LS members enter the study through birth and immigration and existing members leave through death and emigration, but their data is retained[14]. The LS is representative of the whole population of England and Wales, including those in non-private households. The LS is minimal bias due to non-response or attrition, as census coverage is good and rates of linkage high. The high tracing rates contribute to the high linkage rate of LS-members from census to census (88% 2001 to 2011).[15] Response rates to the 2011 Census were very high relative to other national censuses and sample surveys and cohort and panel studies at 94%.[16]

Adults with post age 18 qualifications were asked the titles, subjects, awarding institutions and year. The 1991 graduates include anyone with a degree prior to the 1991 Census. We have restricted this sample to those born before 1975 and survived to 2011 Census. The qualifications were grouped as part of Census data process in 1991 by ONS into 111 subjects. The authors grouped 110 subject areas into four 2021 REF main panel subject areas: A (Life Sciences); B (Physical Sciences); C (Social Sciences); D (Humanities); with clinical medicine removed for the basis of this analysis from life sciences and coded as a separate category.[17] The vast majority of graduates had one degree only. However, a small proportion had multiple degrees and of these a small number of people were recoded as having a degree in clinical medicine based on later qualifications.

Work status variables were collected at the 2011 Census and used to adjust for a proxy for income as this is not collected in the Census. Respondents completed a tick box of options used to determine their participation in paid work the labour market in the week preceding each census. Working status in 2011 with those respondents considered to be 'in work' (this included working, on temporary sick leave, maternity leave, holiday or about to take up a job) with occupational social class based on the National Statistics Socio-economic classification (NS-SEC) with eight categories was used as risk factor for analysis. The categories were: Higher managerial, administrative and professional occupations; Lower managerial, administrative and professional occupations; Intermediate occupations; Small employers and own account workers; Lower supervisory and technical occupations; Semi-routine occupations; Routine occupations; Never worked and long-term unemployed (with missing added).

Demographic and socioeconomic indicators in 2011 were included as potential covariates. Demographic variables included age and age-squared. The results are presented separately by sex.

For peer review only

Respondents were asked about self-rated health 'over the last 12 months: would you say your health has on the whole been: good, fairly good or not good?' The outcome measure was good health compared to poor (fairly good and not good health combined).

Patient and Public Involvement

This research was done without patient involvement. Patients were not invited to comment on the study design and were not consulted to develop patient relevant outcomes or interpret the results. Patients were not invited to contribute to the writing or editing of this document for readability or accuracy.

Results

Table 1 Sample characteristics

Cohort born before 1976 and survived until 2011 Census Completion		NS-SEC			
	1. Higher occupations	2. Intermediate occupations	3. Lower occupations/none	% 1. Higher occupations	Mean Age
Men					
D (Humanities)	573	960	273	32	60
A (Life Sciences)	701	408	120	57	60
B (Physical Sciences)	2853	1837	607	54	61
C (Social Sciences)	2212	1758	479	50	61
Clinical medicine	526	26	15	93	64
Missing degree subject	18	14	11	42	60
Women					
D (Humanities)	463	1877	398	17	58
A (Life Sciences)	478	540	99	43	57
B (Physical Sciences)	302	519	85	33	56
C (Social Sciences)	844	1882	300	28	56
Clinical medicine	278	26	11	88	61
Missing degree subject	supressed	supressed	supressed	supressed	53

Data Source ONS LS; analysis author's own

Table 2 Odds of having good health in 2011 by degree status in 1991. Cohort born before 1975.

	Men				Women			
	OR	p-value	Confidence	Interval	OR	p-value	Confidence	Interval
Has no degree in 1991	1.00				1.00			
Has a degree in 1991	1.86	<0.001	1.77	1.97	1.72	<0.001	1.61	1.84

Adjusted for Age, Age-squared and socio-economic status (NS-SEC)

For peer review only

Data Source ONS LS; analysis author's own

Table 3 Odds of having poor health in 2011 by degree attained by 1991 Cohort born before 1975.

Subject grouped 2021 REF Panel classes (A-C) compared to Humanities (D)

	Men				Women			
Subject	OR	p-value	Confidence	Interval	OR	p-value	Confidence	Interval
D (Humanities)	1.00				1.00			
A (Life Sciences)	0.81	0.088	0.64	1.03	0.84	0.248	0.62	1.13
B (Physical Sciences)	0.85	0.055	0.71	1.00	1.06	0.707	0.77	1.47
C (Social Sciences)	0.84	0.049	0.71	1.00	0.90	0.351	0.72	1.12
Clinical medicine	0.68	0.007	0.52	0.90	0.82	0.395	0.52	1.29
Missing degree subject	0.83	0.658	0.36	1.91	2.07	0.254	0.59	7.24

Adjusted for Age, Age-squared and socio-economic status (NS-SEC)

Data Source ONS LS; analysis author's own

The majority of clinical medicine graduates (93% of men and 88% of women) are employed in higher occupational classifications. This is compared with 32% of male graduates in humanities graduates and 17% of female (Table 1). The mean age of female graduates is lower than that of male by 2-7 years depending on degree subject (Table 1).

Male graduates had 86% higher odds of having good health than male non-graduates after adjustment for age and socio-economic position (confidence intervals 1.77-1.97). Female graduates had 72% higher odds of having good health than female non-graduates after adjustment for age and socio-economic position (confidence intervals 1.61-1.84) (Table 2).

Male clinical medicine graduates had 32% lower odds of having poor health than humanities graduates after adjustment for age and socio-economic position (confidence intervals 0.52-0.90). Male social sciences graduates also had lower odds of having poor health than humanities graduates after adjustment for age and socio-economic position but these were marginally significant, but life sciences and physical science graduates did not. There were no significant differences by degree subject for women (Table 3).

Conclusions: Male graduates in clinical medicine have lower odds of poor self-reported health. Knowledge of medicine may confer a health advantage for men above that of other degrees. Both in that the study of medicine may inform personal health behaviour decisions and earlier diagnosis through skills in research of clinical information and from knowing other experts in the medical field. Financial benefits of studying medicine have also been cited. Ross and Wu[18] found that fulfilling work and high income were very important in explaining the education-health link. As we found in previous work[8] medicine is one of the degree subjects which increases earnings the most - not only much more than the humanities and social science degrees, but also more than other sciences, and hence this could explain some of its strong positive impact of health. The census does not however collect details of income. The vast majority of clinical medicine graduates are employed in higher occupations.

For peer review only

Why these benefits were only experienced by men might be explained by higher salaries of male clinicians or by the benefits of health-related knowledge mediating the gender differentials in poorer health behaviours. There may also be selection bias, with men being more likely admitted to medical school and more likely to pursue a career in science generally than women, with men more likely to get employment and stay in a medical professions than women, and with men's careers less likely to be affected by family and childbearing responsibilities (though as this study looks at education rather than occupation the latter may be less of an issue). The mean age of the women in the sample is slightly younger than the men. Cutler and Lleras-Muney[19] found that specific factual knowledge, e.g. on the harms of smoking and drinking, accounts for around 10% of the education gradient in health behaviours. We would obviously expect this specific factual knowledge to be highest for clinical medicine graduates. This could be further investigated by studying other graduates with health-related qualifications. The Medical Schools Selection Alliance details a minimum of three A levels (post-16) with qualifications usually in lab based sciences and often a third science subject for application to study medicine in the UK.[20] There are no post-16 academic subjects explicitly covering human health other than vocational and technical qualifications in Health and Social Care,[21] with Human Biology A level phased out in 2017.[22] Personal, social, health and economic education (PSHE) is a non-statutory subject on the English school curriculum in maintained schools and academies to age 16, though all state schools should make provision for its teaching.[23] Whether a compulsory GCSE and optional A level in a health related discipline would improve the population's health remains open for debate and to persuade medical schools whether this would form part of a suitable suite of qualifications with which to apply to medical school would also be challenging. This study has looked at graduates of medicine rather than those practising medicine. It is beyond the scope of the paper to look at how these outcomes may differ for those who study medicine but are employed in other fields though this is a potential area for future research, and gender may play an interesting role here.

CeLSIUS is supported by the ESRC (Award Ref: ES/R00823X/1) and therefore their service is free to academic and public sector researchers in most circumstances. This work contains statistical data from ONS which is Crown Copyright. The use of the ONS statistical data in this work does not imply the endorsement of the ONS in relation to the interpretation or analysis of the statistical data. This work uses research datasets which may not exactly reproduce National Statistics aggregates. Data is available to approved researchers.

1 Freedman V, Martin LG, "The role of education in explaining and forecasting trends in functional limitations among older Americans." Demography, 1999; **36**(4): 461-473

2 Elo IT, Preston SH. Educational differentials in mortality: United States, 1979-85. Soc Sci Med, 1996;**42**(1):47-57.

3 Valkonen T. Adult mortality and level of education: a comparison of six countries. In: John Fox, ed. Health Inequalities in European Countries. Aldershot: Gower Publishing Company Limited 1989: 142-162.

4 Walsemann KM, Bell BA, Hummer RA. Effects of Timing and Level of Degree Attained on Depressive Symptoms and Self-Rated Health at Midlife. Am J Public Health. 2012; **102**(3): 557–563.

5 Rogers RG , Everett BG, Zajacova A, Hummer RA. Educational Degrees and Adult Mortality Risk in the United States. Biodemography Soc Biol. 2010; 56(1): 80–99.

For peer review only

6 Kasey Buckles , Andreas Hagemann, Ofer Malamud Melinda Morrill Abigail Wozniak The effect of college education on mortality Journal of Health Economics 2016; **50**: 99–114

7 Bolton P. Education: Historical statistics Standard Note: SN/SG/4252 Social & General Statistics HoC Library, 2012.

8 Belfield C, Britton J, Buscha F, Dearden L, Dickson M, van der Erve L, Sibieta L, Vignoles A, Walker I, Zhu Y. The impact of undergraduate degrees on early-career earnings Institute for Fiscal Studies Department for Education 2018. https://assets.publishing.service.gov.uk/government/uploads/system/uploads/attachment_data/file/759278/The_impact_of_undergraduate_degrees_on_early-career_earnings.pdf

9 Chevalier A. Forschungsinstitut zur Zukunft der Arbeit Subject Choice and Earnings of UK Graduates IZA DP No. 5652 2011.

10 Heckman JJ, Humphries JE, Veramendi G, "Returns to Education: The Causal Effects of Education on Earnings, Health, and Smoking," Journal of Political Economy, University of Chicago Press, 2018; **126**(S1):197-246

11 Fletcher JM, Frisvold DE. Higher Education and Health Investments: Does More Schooling Affect Preventive Health Care Use? Journal of Human Capital 2009 **3**:2, 144-176

12 Belfield C, Britton J, van der Erve L, Shephard N. Where is the money going? Estimating the government cost of different university degrees IFS Briefing Note BN244 2019

13 ONS Graduates in the UK Labour Market: 2013 https://www.ons.gov.uk/employmentandlabourmarket/peopleinwork/employmentandemployeetypes/articles/graduatesintheuklabourmarket/2013-11-19

14 Shelton N, Marshall CE, Stuchbury R, Grundy E, Dennett A, Tomlinson J, Duke-Williams, Xun W (2019) Cohort Profile: the Office for National Statistics Longitudinal Study (The LS) International Journal of Epidemiology, 2019;**48**(2)383–384g,

15 Lynch K, Leib S, Warren J, Rogers N, Buxton J, Longitudinal Study 2001 – 2011 Completeness of census linkage Series LS No. 11 https://www.ons.gov.uk/file?uri=/aboutus/whatwedo/paidservices/longitudinalstudyls/longitudinalstudy20012011completenessofcensuslinkage.pdf (6 February 2018, date last accessed).

16 Office for National Statistics. Response Rates in the 2011 Census. Titchfield: Office for National Statistics, 2012. file:///C:/Users/stinks/AppData/Local/Temp/responseratesinthe2011censusv2dec2012final_tcm77-271600.pdf (6 February 2018, date last accessed).

17 UKRI Units of assessment 2019. https://www.ref.ac.uk/panels/units-of-assessment/ (6 February 2018, date last accessed).

18 Ross CE, Wu C-L. The Links Between Education and Health American Sociological Review 1995; **60** (5)719-745

19 Cutler and Adriana Lleras-Muney Understanding Differences in Health Behaviors by Education J Health Econ. 2010;**29**(1): 1–28.

20 Medical Schools Council Selection Alliance (2018) Entry requirements for UK medical schools 2018 entry https://www.medschools.ac.uk/media/2032/msc-entry-requirements-for-uk-medical-schools.pdf (6 February 2018, date last accessed).

21 UCAS (2018) UCAS Tariff tables Tariff points for entry to higher education from 2019 https://www.ucas.com/file/63536/download?token=lKi4qZse (6 February 2018, date last accessed).

22 Ofqual (2015) Further Decisions for Completing GCSE, AS and A Level Reform in 2017 https://assets.publishing.service.gov.uk/government/uploads/system/uploads/attachment_data/file/479635/2015-05-14-reform-of-gcses-as-and-a-levels-in-2017-may-2015.pdf (6 February 2018, date last accessed).

23 DfE (2019) Personal, social, health and economic (PSHE) education https://www.gov.uk/government/publications/personal-social-health-and-economic-education-pshe/personal-social-health-and-economic-pshe-education (6 February 2018, date last accessed).

For peer review only

STROBE Statement—checklist of items that should be included in reports of observational studies

	Item No.	Recommendation	Page No.
**Title and abstract**	1	(*a*) Indicate the study's design with a commonly used term in the title or the abstract	1
		(*b*) Provide in the abstract an informative and balanced summary of what was done and what was found	2
**Introduction**			
Background/rationale	2	Explain the scientific background and rationale for the investigation being reported	3-4
Objectives	3	State specific objectives, including any prespecified hypotheses	5
**Methods**			
Study design	4	Present key elements of study design early in the paper	5
Setting	5	Describe the setting, locations, and relevant dates, including periods of recruitment, exposure, follow-up, and data collection	5
Participants	6	(*a*) *Cohort study*—Give the eligibility criteria, and the sources and methods of selection of participants. Describe methods of follow-up<br>*Case-control study*—Give the eligibility criteria, and the sources and methods of case ascertainment and control selection. Give the rationale for the choice of cases and controls<br>*Cross-sectional study*—Give the eligibility criteria, and the sources and methods of selection of participants	5
		(*b*) *Cohort study*—For matched studies, give matching criteria and number of exposed and unexposed<br>*Case-control study*—For matched studies, give matching criteria and the number of controls per case	5
Variables	7	Clearly define all outcomes, exposures, predictors, potential confounders, and effect modifiers. Give diagnostic criteria, if applicable	5
Data sources/ measurement	8*	For each variable of interest, give sources of data and details of methods of assessment (measurement). Describe comparability of assessment methods if there is more than one group	5
Bias	9	Describe any efforts to address potential sources of bias	5
Study size	10	Explain how the study size was arrived at	5

Continued on next page

For peer review only

Quantitative variables	11	Explain how quantitative variables were handled in the analyses. If applicable, describe which groupings were chosen and why	5
Statistical methods	12	(*a*) Describe all statistical methods, including those used to control for confounding	5
		(*b*) Describe any methods used to examine subgroups and interactions	5
		(*c*) Explain how missing data were addressed	
		(*d*) *Cohort study*—If applicable, explain how loss to follow-up was addressed<br>*Case-control study*—If applicable, explain how matching of cases and controls was addressed<br>*Cross-sectional study*—If applicable, describe analytical methods taking account of sampling strategy	
		(*e*) Describe any sensitivity analyses	
**Results**			
Participants	13*	(a) Report numbers of individuals at each stage of study—eg numbers potentially eligible, examined for eligibility, confirmed eligible, included in the study, completing follow-up, and analysed	6
		(b) Give reasons for non-participation at each stage	
		(c) Consider use of a flow diagram	
Descriptive data	14*	(a) Give characteristics of study participants (eg demographic, clinical, social) and information on exposures and potential confounders	6
		(b) Indicate number of participants with missing data for each variable of interest	6
		(c) *Cohort study*—Summarise follow-up time (eg, average and total amount)	6
Outcome data	15*	*Cohort study*—Report numbers of outcome events or summary measures over time	*6*
		Case-control study—Report numbers in each exposure category, or summary measures of exposure	*6*
		Cross-sectional study—Report numbers of outcome events or summary measures	
Main results	16	(*a*) Give unadjusted estimates and, if applicable, confounder-adjusted estimates and their precision (eg, 95% confidence interval). Make clear which confounders were adjusted for and why they were included	6
		(*b*) Report category boundaries when continuous variables were categorized	
		(*c*) If relevant, consider translating estimates of relative risk into absolute risk for a meaningful time period	

Continued on next page

For peer review only

Other analyses	17	Report other analyses done—eg analyses of subgroups and interactions, and sensitivity analyses	
**Discussion**			
Key results	18	Summarise key results with reference to study objectives	7-8
Limitations	19	Discuss limitations of the study, taking into account sources of potential bias or imprecision. Discuss both direction and magnitude of any potential bias	7-8
Interpretation	20	Give a cautious overall interpretation of results considering objectives, limitations, multiplicity of analyses, results from similar studies, and other relevant evidence	7-8
Generalisability	21	Discuss the generalisability (external validity) of the study results	7-8
**Other information**			
Funding	22	Give the source of funding and the role of the funders for the present study and, if applicable, for the original study on which the present article is based	8

*Give information separately for cases and controls in case-control studies and, if applicable, for exposed and unexposed groups in cohort and cross-sectional studies.

Note: An Explanation and Elaboration article discusses each checklist item and gives methodological background and published examples of transparent reporting. The STROBE checklist is best used in conjunction with this article (freely available on the Web sites of PLoS Medicine at http://www.plosmedicine.org/, Annals of Internal Medicine at http://www.annals.org/, and Epidemiology at http://www.epidem.com/). Information on the STROBE Initiative is available at www.strobe-statement.org.

For peer review only

For peer review only - http://bmjopen.bmj.com/site/about/guidelines.xhtml

Is studying medicine good for your health? Long term health outcomes of a cohort of clinical medicine graduates in England and Wales in the ONS Longitudinal Study.

Journal:	*BMJ Open*
Manuscript ID	bmjopen-2020-041224.R1
Article Type:	Research
Date Submitted by the Author:	25-Nov-2020
Complete List of Authors:	Shelton, Nicola; UCL, Department of Epidemiology and Public Health<br>Duke-Williams, Oliver; UCL, Department of Information Studies<br>van de Erve, Laura; Institute for Fiscal Studies<br>Britton, Jack; Institute for Fiscal Studies<br>Xun, Wei; University College London Institute of Epidemiology and Health Care
**Primary Subject Heading**:	Epidemiology
Secondary Subject Heading:	Medical education and training
Keywords:	MEDICAL EDUCATION & TRAINING, EPIDEMIOLOGY, PUBLIC HEALTH, EDUCATION & TRAINING (see Medical Education & Training)

I, the Submitting Author has the right to grant and does grant on behalf of all authors of the Work (as defined in the below author licence), an exclusive licence and/or a non-exclusive licence for contributions from authors who are: i) UK Crown employees; ii) where BMJ has agreed a CC-BY licence shall apply, and/or iii) in accordance with the terms applicable for US Federal Government officers or employees acting as part of their official duties; on a worldwide, perpetual, irrevocable, royalty-free basis to BMJ Publishing Group Ltd ("BMJ") its licensees and where the relevant Journal is co-owned by BMJ to the co-owners of the Journal, to publish the Work in this journal and any other BMJ products and to exploit all rights, as set out in our licence.

The Submitting Author accepts and understands that any supply made under these terms is made by BMJ to the Submitting Author unless you are acting as an employee on behalf of your employer or a postgraduate student of an affiliated institution which is paying any applicable article publishing charge ("APC") for Open Access articles. Where the Submitting Author wishes to make the Work available on an Open Access basis (and intends to pay the relevant APC), the terms of reuse of such Open Access shall be governed by a Creative Commons licence – details of these licences and which Creative Commons licence will apply to this Work are set out in our licence referred to above.

Other than as permitted in any relevant BMJ Author's Self Archiving Policies, I confirm this Work has not been accepted for publication elsewhere, is not being considered for publication elsewhere and does not duplicate material already published. I confirm all authors consent to publication of this Work and authorise the granting of this licence.

For peer review only

Is studying medicine good for your health? Long term health outcomes of a cohort of clinical medicine graduates in England and Wales in the ONS Longitudinal Study

Nicola Shelton[1]* Oliver Duke-Williams[2] Laura van der Erve[3] Jack Britton[3] Wei Xun[1]

1 Centre for Longitudinal Study Information and User Support (CeLSIUS), Department of Epidemiology and Public Health, UCL, London, UK.

2 Department of Information Studies, UCL, London, UK.

3. The Institute for Fiscal Studies, London, UK.

*Corresponding Author: Centre for Longitudinal Study Information and User Support (CeLSIUS), Department of Epidemiology and Public Health, UCL, 1-19 Torrington Place, London WC1E 6BT, UK. Email: n.shelton@ucl.ac.uk Tel +442076795648

Word count, excluding title page, abstract, strengths and limitations and references, figures and tables

2404

For peer review only

Abstract

Objective: to quantify the protective effect on health associated with study of a clinical medicine degree.

Design: Prospective population based cohort data collected at census and linked over time study: cohort born before 1976 and survived to 2011, Subgroup analysis on those who reported having a degree at 1991 census.

Setting: England and Wales population based including institutions.

Participants: 159,116men 174,062women. 13,390 men with degrees 8,143 women with degrees.

Main outcome measure: self-reported general health in 2011 based on logistic regression analysis.

Results: Male graduates had 92% higher odds of having good or very good health than male non-graduates after adjustment for age and socio-economic position (confidence intervals 1.82-2.03). Female graduates had 72% higher odds of having good or very good health than female non-graduates after adjustment for age and socio-economic position (confidence intervals 1.73-1.84).

Male clinical medicine graduates had 45% higher odds of having good or very good health than male humanities graduates after adjustment for age and socio-economic position (confidence intervals 1.09--1.92). Male physical sciences graduates also had higher odds of having good or very good health than male humanities graduates after adjustment for age and socio-economic position, but life sciences and social science graduates did not. There were no significant differences by degree subject for women.

Conclusions: Male graduates in clinical medicine have higher odds of good self-reported health. Knowledge of medicine may confer a health advantage for men above that of other degrees.

Strengths and Limitations of this Study

Large sample size (total N > 1 000 000 in full panel). This is by far the largest nationally representative longitudinal dataset in the UK.

Length of follow-up available (40 years, 1971–2011 for main census data) with life events for ONS Longitudinal Study members available until about 2 years before the current year of analysis.

The high tracing rates contribute to the high linkage rate of LS members from census to census (88% 2001 to 2011).

No behavioural or income data collected.

Census is every 10 years, so updates are limited.

For peer review only

Introduction

Several studies have shown the health benefits of education and the gradient in this. Education to degree level confers a greater health advantage.

Freedman and Martin[1] found that education level accounted for declines in functional limitations among older Americans from 1984 to 1993, and high school graduate education was the most important in accounting for recent trends of the eight demographic and socioeconomic variables they considered.

Elo and Preston[2] found proportionate reductions in mortality for each one-year increase in schooling in the United States at ages 35-54 comparable to those estimated for a number of European countries by Valkonen[3]. The main difference they found between the United States and Europe was that in the U.S. mortality reduction with years of schooling was quite similar for both men and women, while in a number of European countries male mortality was more greatly reduced than female mortality with educational attainment.

Walseman et al[4] had explored if later life qualifications benefited health. Among respondents with no degree, a high school diploma, or a post–high school certificate at 25 years of age, attaining at least a bachelor's degree by midlife was associated with fewer depressive symptoms and better self-rated health at midlife compared with respondents who did not attain a higher degree by midlife.

Rogers et al[5] showed that educational degrees were associated with reduced mortality risk in three cohorts of U.S adults aged 25 and above in 1997–2002 though they showed more marked gender differences, with associations not significant in older women and were weaker in women than in men. Among males in all cohort groups, there were gradients by educational degree level in the risk of death. The overall educational degree gradient was evident in all cohorts of women, although the mortality advantages for those with postsecondary degrees were generally not as pronounced among women as among men.

More recently Buckles[6] found that college (university) education among white men born between 1942 and 1953 in the US was associated with lower mortality and higher earning, but also with higher levels of health insurance offering a pathway to better health outcomes in the US but not in the UK, but also greater reductions in smoking and higher levels of physical activity.

We highlight the role of education for several reasons. First, education is strongly associated with many health- related behaviours over the life cycle, which frequently are not measured directly in nationally representative surveys and administrative data. Further, unlike some other measures of socioeconomic status such as occupation and income, educational qualification data are straightforward to report, and are generally fixed for each individual relatively early in life. In addition, Higher Education participation in UK increased from 3.4% in 1950, to 8.4% in 1970, to 19.3% in 1990. [7]

There has also been considerable work looking at the earnings returns to different degree subjects,[8-9] and also work looking at the wider returns to attending higher education, including the health outcomes.[10-11] However, there has been very little investigation in the health returns by different degree subjects. The UK government is increasingly focussed on the returns to different degrees as government subsidies of different subject areas has increased significantly;[12] understanding these wider returns is therefore highly important.

For peer review only

The research looks at the health outcomes of adults by which degree they studied. Self-rated health correlates strongly with clinical assessments of morbidity and subsequent mortality in many studies and in the ONS Longitudinal Study specifically a strong association has been shown between reporting of fairly good health or not good health combined with mortality (Young et al, 2010)[13]. The hypothesis was that clinical medicine would confer health advantage. Graduates in humanities have lower salaries and lower employment rates in the UK than graduates in medicine and science (ONS, 2013)[14] yet there are no studies of how this related to health outcomes. This is the first study to consider the health benefits of studying different degree subjects.

We hypothesise that clinical medicine graduates will have higher odds of good or very good self-reported health compared with fair, bed or very bad health combined.

Methods

The ONS Longitudinal Study (LS) comprises people born on one of four selected dates of birth and so makes up about 1% of the total population in England and Wales – that data is linked for five successive censuses starting at 1971; new LS members enter the study through birth and immigration and existing members leave through death and emigration, but their data is retained[15]. The LS is representative of the whole population of England and Wales, including those in non-private households. The LS is minimal bias due to non-response or attrition, as census coverage is good and rates of linkage high. The high tracing rates contribute to the high linkage rate of LS-members from census to census (88% 2001 to 2011).[16] Response rates to the 2011 Census were very high relative to other national censuses and sample surveys and cohort and panel studies at 94%.[17]

Adults with post age 18 qualifications were asked the titles, subjects, awarding institutions and year. The 1991 graduates include anyone with a degree prior to the 1991 Census. We have restricted this sample to those born before 1976 (to exclude children who may have been erroneously assigned a higher education qualification) and survived to 2011 Census. The qualifications were grouped as part of Census data process in 1991 by ONS into 111 subjects. The authors grouped 110 subject areas into four 2021 REF main panel subject areas: A (Life Sciences); B (Physical Sciences); C (Social Sciences); D (Humanities); with clinical medicine removed for the basis of this analysis from life sciences and coded as a separate category.[18] The vast majority of graduates had one degree only. However, a small proportion had multiple degrees and of these a small number of people were recoded as having a degree in clinical medicine based on later qualifications. All other graduates were coded by their first degree awarded prior to the 1991 Census. Degrees awarded after 1991 by subject were not considered as this question was not asked in subsequent censuses.

Work status variables were collected at the 2011 Census and used to adjust for a proxy for income as this is not collected in the Census. Respondents completed a tick box of options used to determine their participation in paid work the labour market in the week preceding each census. Working status in 2011 with those respondents considered to be 'in work' (this included working, on temporary sick leave, maternity leave, holiday or about to take up a job) with occupational social class based on the National Statistics Socio-economic classification (NS-SEC) with three categories was used as risk factor for analysis. The categories were: Managerial, administrative, and professional occupations; Intermediate occupations Routine and manual occupations and never worked and unemployed combined.

For peer review only

Demographic and socioeconomic indicators in 2011 were included as potential covariates. Demographic variables included age and age-squared. The results are presented separately by sex.

Respondents were asked about self-rated health 'How is your health in general?' The outcome measure was good health and very good health combined compared to poor (fair, bad, and very bad health combined).

Patient and Public Involvement

This research was done without patient involvement. Patients were not invited to comment on the study design and were not consulted to develop patient relevant outcomes or interpret the results. Patients were not invited to contribute to the writing or editing of this document for readability or accuracy.

Results

Table 1 Sample characteristics

Cohort born before 1976 and survived until 2011 Census Completion who reported having a higher education degree in 1991 Census by NS-SEC and mean age

	1. Higher occupations	2. Intermediate occupations	3. Lower occupations /none	% 1. Higher occupations	Mean Age
Men					
D (Humanities)	573	960	251	32	62
A (Life Sciences)	701	408	115	57	61
B (Physical Sciences)	2853	1837	553	54	62
C (Social Sciences)	2212	1758	432	50	62
Clinical medicine	526	26	12	93	66
Missing degree subject	18	14	10	43	61
Women					
D (Humanities)	463	1877	347	17	60
A (Life Sciences)	478	540	88	43	58
B (Physical Sciences)	302	519	74	34	56
C (Social Sciences)	844	1882	261	28	57
Clinical medicine	278	26	10	89	62
Missing degree subject	supressed	supressed	supressed	supressed	54

Data Source ONS LS; analysis author's own

Table 2 Odds of having good or very good health in 2011 by degree status in 1991. Cohort born before 1976.

	Men	n=159,116			Women	n= 174,062		
	OR	p-value	Confidence	Interval	OR	p-value	Confidence	Interval
Does not have a degree in 1991	1.00				1.00			
Has a degree in 1991	1.92	<0.001	1.82	2.03	1.85	<0.001	1.73	1.98

Adjusted for Age, Age-squared and socio-economic status (NS-SEC); constant not shown

Data Source ONS LS; analysis author's own

Table 3 Odds of having good or very good health in 2011 by degree attained by 1991 Cohort born before 1976.

Subject grouped 2021 REF Panel classes (A-C) compared to Humanities (D)

	Men	n=13,390			Women	n= 8,143		
Subject	OR	p-value	Confidence	Interval	OR	p-value	Confidence	Interval
D (Humanities)	1.00				1.00			
A (Life Sciences)	1.16	0.163	0.94	1.44	0.95	0.663	0.77	1.18
B (Physical Sciences)	1.24	0.006	1.06	1.44	0.82	0.086	0.66	1.03
C (Social Sciences)	1.07	0.371	0.92	1.25	0.89	0.140	0.76	1.04
Clinical medicine	1.45	0.011	1.09	1.92	1.10	0.605	0.76	1.60
Missing degree subject	1.06	0.889	0.46	2.48	0.86	0.773	0.32	2.32

Adjusted for Age, Age-squared and socio-economic status (NS-SEC); constant not shown

Data Source ONS LS; analysis author's own

The majority of clinical medicine graduates (93% of men and 89% of women) were employed in higher occupational classifications. This is compared with 32% of male graduates in humanities subjects and 17% of female (Table 1). There were small differences between the mean age group of the groups of graduates analysed. The mean age of male and female clinical medicine graduates in the sample was higher than that of other male and female graduate groups respectively. The mean age of female graduates was lower than that of male by 2-7 years depending on degree subject (Table 1).

Male graduates had 92% higher odds of having good or very good health than male non-graduates after adjustment for age and socio-economic position (confidence intervals 1.82-2.03). Female graduates had 85% higher odds of having good or very good health than female non-graduates after adjustment for age and socio-economic position (confidence intervals 1.73-1.98) (Table 2).

Male clinical medicine graduates had 45% higher odds of having good or very good health than humanities graduates after adjustment for age and socio-economic position (confidence intervals 1.09.-1.92). Male physical sciences graduates also had higher odds of having good or very good health than humanities graduates after adjustment for age and socio-economic position, , but life sciences and social science graduates did not. There were no significant differences by degree subject for women (Table 3).

For peer review only

Conclusions: Male graduates in clinical medicine have higher odds of good or very good self-reported health. Knowledge of medicine may confer a health advantage for men above that of other degrees. The study of medicine may both inform personal health behaviour decisions and also lead to earlier self diagnosis through skills gained in research of clinical information and from knowing other experts in the medical field to consult. Additionally there are financial benefits of studying medicinethat may explain the health advantage. Ross and Wu[19] found that fulfilling work and high income were very important in explaining the education-health link. As we found in previous work[8] medicine is one of the degree subjects which increases earnings the most - not only much more than the humanities and social science degrees, but also more than other sciences, and hence this could explain some of its strong positive impact of health. (This may also partly explain the association seen in physical sciences graduates).The census does not however collect details of income. The vast majority of clinical medicine graduates were employed in higher level occupations. Given the higher mean age of clinical medicine graduates if age selection were explaining the results we would explain this to reduce rather than increase the size of the association found suggesting this is not the explanation.

Why these benefits were only experienced by men might be explained by higher salaries of male clinicians or by the benefits of health-related knowledge mediating the gender differentials in health behaviours. There may also be selection bias, with men being more likely admitted to medical school and more likely to pursue a career in science generally than women, with men more likely to get employment and stay in a medical profession than women, and with men's careers less likely to be affected by family and childbearing responsibilities (though as this study looks at education rather than occupation the latter may be less of an issue). The mean age of the women in the sample was slightly younger than the men. There could also be effects on health where the educational qualification of the head of household may be more important, especially in households which are not headed by women. Cutler and Lleras-Muney[20] found that specific factual knowledge, e.g. on the harms of smoking and drinking, accounts for around 10% of the education gradient in health behaviours. We would obviously expect this specific factual knowledge to be highest for clinical medicine graduates. This could be further investigated by studying other graduates with health-related qualifications. The Medical Schools Selection Alliance details a minimum of three A levels (post-16) with qualifications usually in lab based sciences and often a third science subject for application to study medicine in the UK.[21] There are no post-16 academic subjects explicitly covering human health other than vocational and technical qualifications in Health and Social Care,[22] with Human Biology A level phased out in 2017.[23] Personal, social, health and economic education (PSHE) is a non-statutory subject on the English school curriculum in maintained schools and academies to age 16, though all state schools should make provision for its teaching.[24] Whether a compulsory GCSE and optional A level in a health related discipline would improve the population's health remains open for debate and to persuade medical schools whether this would form part of a suitable suite of qualifications with which to apply to medical school would also be challenging. This study has looked at graduates of medicine rather than those practising medicine. It is beyond the scope of the paper to look at how these outcomes may differ for those who study medicine but are employed in other fields though this is a potential area for future research, and gender may play an interesting role here. Also disentangling the effects of income might be considered if data on income were in future able to be linked to the ONS LS perhaps as part of administrative based censuses.

For peer review only

The permission of the Office for National Statistics to use the Longitudinal Study is gratefully acknowledged, as is the help provided by staff of the Centre for Longitudinal Study Information & User Support (CeLSIUS). CeLSIUS is supported by the ESRC (Award Refs: ES/V003488/1 and ES/R00823X/1).The authors alone are responsible for the interpretation of the data.

This work contains statistical data from ONS which is Crown Copyright. The use of the ONS statistical data in this work does not imply the endorsement of the ONS in relation to the interpretation or analysis of the statistical data. This work uses research datasets which may not exactly reproduce National Statistics aggregates.Contributorship statement: NS and ODW devised the research idea and NS and ODW and WX collated the data and completed the analysis. LVDE and JB co-wrote the literature review and discussion with NS and ODW and provided comment on the full draft text.

Competing interests: There are no competing interests.

Funding: the authors received no funding for the article. NS and ODW are members of CeLSIUS which is supported by the ESRC (Award Refs: ES/V003488/1 and ES/R00823X/1).

Data availability: The data: Office for National Statistics Longitudinal Study (ONS LS) from which this panel is drawn are available from ONS via the Secure Research Service to approved researchers with approved projects. Information and support for the LS for UK-based prospective and current users from the academic, statutory and voluntary sectors can be obtained from the Centre for Longitudinal Study Information and User Support (CeLSIUS) by emailing Celsius@ucl.ac.uk. All other users should contact the ONS Longitudinal Study Development Team (LSDT): LongitudinalStudy@ons.gov.uk. A step-by-step guide to using the LS is available from the CeLSIUS website www.ucl.ac.uk/celsius

1 Freedman V, Martin LG, "The role of education in explaining and forecasting trends in functional limitations among older Americans." Demography, 1999; **36**(4): 461-473

2 Elo IT, Preston SH. Educational differentials in mortality: United States, 1979-85. Soc Sci Med, 1996;**42**(1):47-57.

3 Valkonen T. Adult mortality and level of education: a comparison of six countries. In: John Fox, ed. Health Inequalities in European Countries. Aldershot: Gower Publishing Company Limited 1989: 142-162.

4 Walsemann KM, Bell BA, Hummer RA. Effects of Timing and Level of Degree Attained on Depressive Symptoms and Self-Rated Health at Midlife. Am J Public Health. 2012; **102**(3): 557–563.

5 Rogers RG , Everett BG, Zajacova A, Hummer RA. Educational Degrees and Adult Mortality Risk in the United States. Biodemography Soc Biol. 2010; 56(1): 80–99.

6 Kasey Buckles , Andreas Hagemann, Ofer Malamud Melinda Morrill Abigail Wozniak The effect of college education on mortality Journal of Health Economics 2016; **50**: 99–114

7 Bolton P. Education: Historical statistics Standard Note: SN/SG/4252 Social & General Statistics HoC Library, 2012.

8 Belfield C, Britton J, Buscha F, Dearden L, Dickson M, van der Erve L, Sibieta L, Vignoles A, Walker I, Zhu Y. The impact of undergraduate degrees on early-career earnings Institute for Fiscal Studies Department for Education 2018.

For peer review only

https://assets.publishing.service.gov.uk/government/uploads/system/uploads/attachment_data/file/759278/The_impact_of_undergraduate_degrees_on_early-career_earnings.pdf

9 Chevalier A. Forschungsinstitut zur Zukunft der Arbeit Subject Choice and Earnings of UK Graduates IZA DP No. 5652 2011.

10 Heckman JJ, Humphries JE, Veramendi G, "Returns to Education: The Causal Effects of Education on Earnings, Health, and Smoking," Journal of Political Economy, University of Chicago Press, 2018; **126**(S1):197-246

11 Fletcher JM, Frisvold DE. Higher Education and Health Investments: Does More Schooling Affect Preventive Health Care Use? Journal of Human Capital 2009 **3**:2, 144-176

12 Belfield C, Britton J, van der Erve L, Shephard N. Where is the money going? Estimating the government cost of different university degrees IFS Briefing Note BN244 2019

13 Young H, Grundy E, O'Reilly D, Boyle P. Self-rated health and mortality in the UK: results from the first comparative analysis of the England and Wales, Scotland and Northern Ireland Longitudinal Studies Population Trends 2010;**139** 11-3514 ONS Graduates in the UK Labour Market: 2013 https://www.ons.gov.uk/employmentandlabourmarket/peopleinwork/employmentandemployeetypes/articles/graduatesintheuklabourmarket/2013-11-19

15 Shelton N, Marshall CE, Stuchbury R, Grundy E, Dennett A, Tomlinson J, Duke-Williams, Xun W (2019) Cohort Profile: the Office for National Statistics Longitudinal Study (The LS) International Journal of Epidemiology, 2019;**48**(2)383–384g,

16 Lynch K, Leib S, Warren J, Rogers N, Buxton J, Longitudinal Study 2001 – 2011 Completeness of census linkage Series LS No. 11 https://www.ons.gov.uk/file?uri=/aboutus/whatwedo/paidservices/longitudinalstudyls/longitudinalstudy20012011completenessofcensuslinkage.pdf (6 February 2018, date last accessed).

17 Office for National Statistics. Response Rates in the 2011 Census. Titchfield: Office for National Statistics, 2012. file:///C:/Users/stinks/AppData/Local/Temp/responseratesinthe2011censusv2dec2012final_tcm77-271600.pdf (6 February 2018, date last accessed).

19 UKRI Units of assessment 2019. https://www.ref.ac.uk/panels/units-of-assessment/ (6 February 2018, date last accessed).

19 Ross CE, Wu C-L. The Links Between Education and Health American Sociological Review 1995; **60** (5)719-745

20 Cutler and Adriana Lleras-Muney Understanding Differences in Health Behaviors by Education J Health Econ. 2010;**29**(1): 1–28.

21 Medical Schools Council Selection Alliance (2018) Entry requirements for UK medical schools 2018 entry https://www.medschools.ac.uk/media/2032/msc-entry-requirements-for-uk-medical-schools.pdf (6 February 2018, date last accessed).

For peer review only

22 UCAS (2018) UCAS Tariff tables Tariff points for entry to higher education from 2019 https://www.ucas.com/file/63536/download?token=lKi4qZse (6 February 2018, date last accessed).

23 Ofqual (2015) Further Decisions for Completing GCSE, AS and A Level Reform in 2017 https://assets.publishing.service.gov.uk/government/uploads/system/uploads/attachment_data/file/479635/2015-05-14-reform-of-gcses-as-and-a-levels-in-2017-may-2015.pdf (6 February 2018, date last accessed).

24 DfE (2019) Personal, social, health and economic (PSHE) education https://www.gov.uk/government/publications/personal-social-health-and-economic-education-pshe/personal-social-health-and-economic-pshe-education (6 February 2018, date last accessed).

For peer review only

STROBE Statement—checklist of items that should be included in reports of observational studies

	Item No.	Recommendation	Page No.
**Title and abstract**	1	(*a*) Indicate the study's design with a commonly used term in the title or the abstract	1
		(*b*) Provide in the abstract an informative and balanced summary of what was done and what was found	2
**Introduction**			
Background/rationale	2	Explain the scientific background and rationale for the investigation being reported	3-4
Objectives	3	State specific objectives, including any prespecified hypotheses	5
**Methods**			
Study design	4	Present key elements of study design early in the paper	5
Setting	5	Describe the setting, locations, and relevant dates, including periods of recruitment, exposure, follow-up, and data collection	5
Participants	6	(*a*) *Cohort study*—Give the eligibility criteria, and the sources and methods of selection of participants. Describe methods of follow-up<br>*Case-control study*—Give the eligibility criteria, and the sources and methods of case ascertainment and control selection. Give the rationale for the choice of cases and controls<br>*Cross-sectional study*—Give the eligibility criteria, and the sources and methods of selection of participants	5
		(*b*) *Cohort study*—For matched studies, give matching criteria and number of exposed and unexposed<br>*Case-control study*—For matched studies, give matching criteria and the number of controls per case	5
Variables	7	Clearly define all outcomes, exposures, predictors, potential confounders, and effect modifiers. Give diagnostic criteria, if applicable	5
Data sources/ measurement	8*	For each variable of interest, give sources of data and details of methods of assessment (measurement). Describe comparability of assessment methods if there is more than one group	5
Bias	9	Describe any efforts to address potential sources of bias	5
Study size	10	Explain how the study size was arrived at	5

Continued on next page

For peer review only

Quantitative variables	11	Explain how quantitative variables were handled in the analyses. If applicable, describe which groupings were chosen and why	5
Statistical methods	12	(*a*) Describe all statistical methods, including those used to control for confounding	5
		(*b*) Describe any methods used to examine subgroups and interactions	5
		(*c*) Explain how missing data were addressed	
		(*d*) *Cohort study*—If applicable, explain how loss to follow-up was addressed<br>*Case-control study*—If applicable, explain how matching of cases and controls was addressed<br>*Cross-sectional study*—If applicable, describe analytical methods taking account of sampling strategy	
		(*e*) Describe any sensitivity analyses	
**Results**			
Participants	13*	(a) Report numbers of individuals at each stage of study—eg numbers potentially eligible, examined for eligibility, confirmed eligible, included in the study, completing follow-up, and analysed	6
		(b) Give reasons for non-participation at each stage	
		(c) Consider use of a flow diagram	
Descriptive data	14*	(a) Give characteristics of study participants (eg demographic, clinical, social) and information on exposures and potential confounders	6
		(b) Indicate number of participants with missing data for each variable of interest	6
		(c) *Cohort study*—Summarise follow-up time (eg, average and total amount)	6
Outcome data	15*	*Cohort study*—Report numbers of outcome events or summary measures over time	*6*
		Case-control study—Report numbers in each exposure category, or summary measures of exposure	*6*
		Cross-sectional study—Report numbers of outcome events or summary measures	
Main results	16	(*a*) Give unadjusted estimates and, if applicable, confounder-adjusted estimates and their precision (eg, 95% confidence interval). Make clear which confounders were adjusted for and why they were included	6
		(*b*) Report category boundaries when continuous variables were categorized	
		(*c*) If relevant, consider translating estimates of relative risk into absolute risk for a meaningful time period	

Continued on next page

For peer review only

Other analyses	17	Report other analyses done—eg analyses of subgroups and interactions, and sensitivity analyses	
**Discussion**			
Key results	18	Summarise key results with reference to study objectives	7-8
Limitations	19	Discuss limitations of the study, taking into account sources of potential bias or imprecision. Discuss both direction and magnitude of any potential bias	7-8
Interpretation	20	Give a cautious overall interpretation of results considering objectives, limitations, multiplicity of analyses, results from similar studies, and other relevant evidence	7-8
Generalisability	21	Discuss the generalisability (external validity) of the study results	7-8
**Other information**			
Funding	22	Give the source of funding and the role of the funders for the present study and, if applicable, for the original study on which the present article is based	8

*Give information separately for cases and controls in case-control studies and, if applicable, for exposed and unexposed groups in cohort and cross-sectional studies.

Note: An Explanation and Elaboration article discusses each checklist item and gives methodological background and published examples of transparent reporting. The STROBE checklist is best used in conjunction with this article (freely available on the Web sites of PLoS Medicine at http://www.plosmedicine.org/, Annals of Internal Medicine at http://www.annals.org/, and Epidemiology at http://www.epidem.com/). Information on the STROBE Initiative is available at www.strobe-statement.org.

For peer review only

BMJ Open

Is studying medicine good for your health? Long term health outcomes of a cohort of clinical medicine graduates in England and Wales in the ONS Longitudinal Study.

Journal:	*BMJ Open*
Manuscript ID	bmjopen-2020-041224.R2
Article Type:	Research
Date Submitted by the Author:	12-Jan-2021
Complete List of Authors:	Shelton, Nicola; UCL, Department of Epidemiology and Public Health<br>Duke-Williams, Oliver; UCL, Department of Information Studies<br>van de Erve, Laura; Institute for Fiscal Studies<br>Britton, Jack; Institute for Fiscal Studies<br>Xun, Wei; University College London Institute of Epidemiology and Health Care
**Primary Subject Heading**:	Epidemiology
Secondary Subject Heading:	Medical education and training
Keywords:	MEDICAL EDUCATION & TRAINING, EPIDEMIOLOGY, PUBLIC HEALTH, EDUCATION & TRAINING (see Medical Education & Training)

SCHOLARONE™
Manuscripts

I, the Submitting Author has the right to grant and does grant on behalf of all authors of the Work (as defined in the below author licence), an exclusive licence and/or a non-exclusive licence for contributions from authors who are: i) UK Crown employees; ii) where BMJ has agreed a CC-BY licence shall apply, and/or iii) in accordance with the terms applicable for US Federal Government officers or employees acting as part of their official duties; on a worldwide, perpetual, irrevocable, royalty-free basis to BMJ Publishing Group Ltd ("BMJ") its licensees and where the relevant Journal is co-owned by BMJ to the co-owners of the Journal, to publish the Work in this journal and any other BMJ products and to exploit all rights, as set out in our licence.

The Submitting Author accepts and understands that any supply made under these terms is made by BMJ to the Submitting Author unless you are acting as an employee on behalf of your employer or a postgraduate student of an affiliated institution which is paying any applicable article publishing charge ("APC") for Open Access articles. Where the Submitting Author wishes to make the Work available on an Open Access basis (and intends to pay the relevant APC), the terms of reuse of such Open Access shall be governed by a Creative Commons licence – details of these licences and which Creative Commons licence will apply to this Work are set out in our licence referred to above.

Other than as permitted in any relevant BMJ Author's Self Archiving Policies, I confirm this Work has not been accepted for publication elsewhere, is not being considered for publication elsewhere and does not duplicate material already published. I confirm all authors consent to publication of this Work and authorise the granting of this licence.

For peer review only

Is studying medicine good for your health? Long term health outcomes of a cohort of clinical medicine graduates in England and Wales in the ONS Longitudinal Study

Nicola Shelton[1]* Oliver Duke-Williams[2] Laura van der Erve[3] Jack Britton[3] Wei Xun[1]

1. Centre for Longitudinal Study Information and User Support (CeLSIUS), Department of Epidemiology and Public Health, UCL, London, UK.

2. Department of Information Studies, UCL, London, UK.

3. The Institute for Fiscal Studies, London, UK.

*Corresponding Author: Centre for Longitudinal Study Information and User Support (CeLSIUS), Department of Epidemiology and Public Health, UCL, 1-19 Torrington Place, London WC1E 6BT, UK. Email: n.shelton@ucl.ac.uk Tel +442076795648

Word count, excluding title page, abstract, strengths and limitations and references, figures and tables

2261

For peer review only

Abstract

Objective: to quantify the potential protective effect on health associated with study of a clinical medicine degree.

Design: Prospective population based cohort data collected at census and linked over time study: cohort born before 1976 and survived to 2011. Subgroup analysis on those who reported having a degree at 1991 census.

Setting: England and Wales population based including institutions.

Participants: 159,116 men 174,062 women. 13,390 men with degrees 8,143 women with degrees.

Main outcome measure: self-reported general health in 2011 based on logistic regression analysis.

Results: Male graduates had 92% higher odds of having good or very good health than male non-graduates after adjustment for age and socio-economic position (confidence intervals 1.82-2.03). Female graduates had 85% higher odds of having good or very good health than female non-graduates after adjustment for age and socio-economic position (confidence intervals 1.73-1.-98).

Male clinical medicine graduates had 45% higher odds of having good or very good health than male humanities graduates after adjustment for age and socio-economic position (confidence intervals 1.09--1.92). Male physical sciences graduates also had higher odds of having good or very good health than male humanities graduates after adjustment for age and socio-economic position, but life sciences and social science graduates did not. There were no significant differences by degree subject for women.

Conclusions: Male graduates in clinical medicine have higher odds of good self-reported health. Knowledge of medicine may confer a health advantage for men above that of other degrees.

Strengths and Limitations of this Study

Large sample size (total N > 1 000 000 in full panel). This is by far the largest nationally representative longitudinal dataset in the UK.

Length of follow-up available (40 years, 1971–2011 for main census data) with life events for ONS Longitudinal Study members available until about 2 years before the current year of analysis.

The high tracing rates contribute to the high linkage rate of LS members from census to census (88% 2001 to 2011).

No behavioural or income data collected.

Census is every 10 years, so updates are limited.

For peer review only

Introduction

Several studies have shown the health benefits of education and the gradient in this. Education to degree level confers a greater health advantage.

Freedman and Martin[1] found that education level accounted for declines in functional limitations among older Americans from 1984 to 1993, and high school graduate education was the most important in accounting for recent trends of the eight demographic and socioeconomic variables they considered.

Elo and Preston[2] found proportionate reductions in mortality for each one-year increase in schooling in the United States at ages 35-54 comparable to those estimated for a number of European countries by Valkonen[3]. The main difference they found between the United States and Europe was that in the U.S. mortality reduction with years of schooling was quite similar for both men and women, while in a number of European countries male mortality was reduced more than female mortality was with educational attainment.

Walseman et al[4] explored if later life qualifications benefited health. Among respondents with no degree, a high school diploma, or a post–high school certificate at 25 years of age, attaining at least a bachelor's degree by midlife was associated with fewer depressive symptoms and better self-rated health at midlife compared with respondents who did not attain a higher degree by midlife.

Rogers et al[5] showed that educational degrees were associated with reduced mortality risk in three cohorts of U.S. adults aged 25 and above in 1997–2002 though they showed more marked gender differences, with associations not significant in older women and weaker in women than in men. Among males in all cohort groups, there were gradients by educational degree level in the risk of death. The overall educational degree gradient was evident in all cohorts of women, although the mortality advantages for those with postsecondary degrees were generally not as pronounced among women as among men.

More recently Buckles[6] found that college (university) education among white men born between 1942 and 1953 in the U.S. was associated with lower mortality and higher earning, and also greater reductions in smoking and higher levels of physical activity. College education was also with higher levels of health insurance offering a pathway to better health outcomes, but more inequality in the US.

We highlight the role of education for several reasons. First, education is strongly associated with many health- related behaviours over the life cycle, which are frequently not measured directly in nationally representative surveys and administrative data. Further, unlike some other measures of socioeconomic status such as occupation and income, educational qualification data are straightforward to report, and are generally fixed for each individual relatively early in life. In addition, Higher Education participation in the UK increased from 3.4% in 1950, to 8.4% in 1970, to 19.3% in 1990. [7]

There has also been considerable work looking at the earnings returns to different degree subjects,[8-9] and also work looking at the wider returns to attending higher education, including the health outcomes.[10-11] However, there has been very little investigation in the health returns by different degree subjects. The UK government is increasingly focussed on the returns to different degrees as government subsidies of different subject areas has increased significantly;[12] understanding these wider returns is therefore highly important.

For peer review only

The research looks at the health outcomes of adults by which degree they studied. Self-rated health correlates strongly with clinical assessments of morbidity and subsequent mortality in many studies and in the ONS Longitudinal Study specifically, a strong association has been shown between reporting of fairly good health and not good health combined, compared with good health, with mortality (Young et al, 2010)[13]. The hypothesis was that clinical medicine would confer health advantage. Graduates in humanities have lower salaries and lower employment rates in the UK than graduates in medicine and science (ONS, 2013)[14] yet there are no studies of how this related to health outcomes. This is the first study to consider the health benefits of studying different degree subjects.

We hypothesise that clinical medicine graduates will have higher odds of good or very good self-reported health compared with fair, bad or very bad health combined.

Methods

The ONS Longitudinal Study (LS) comprises people born on one of four selected dates of birth and so makes up about 1% of the total population in England and Wales. That data is linked for five successive censuses starting at 1971; new LS members enter the study through birth and immigration and existing members leave through death and emigration, but their data is retained.[15] The LS is representative of the whole population of England and Wales, including those in non-private households. The LS has minimal bias due to non-response or attrition, as census coverage is good and rates of linkage high. The high tracing rates contribute to the high linkage rate of LS-members from census to census (88% 2001 to 2011).[16] Response rates to the 2011 Census were very high relative to other national censuses and sample surveys, cohort and panel studies at 94%.[17]

Adults with post age 18 qualifications were asked the titles, subjects, awarding institutions and year in the 1991 census. These pre-1991 graduates include anyone with a degree prior to the 1991 Census. We have restricted this sample to those born before 1976 (to exclude children who may have been erroneously assigned a higher education qualification) and survived to 2011 Census. The qualifications were grouped as part of Census data processing in 1991 by ONS into 111 subjects. The authors grouped 110 subject areas into four 2021 Research Excellence Framework (REF) main panel subject areas: A (Life Sciences); B (Physical Sciences); C (Social Sciences); D (Humanities); with clinical medicine removed for the basis of this analysis from life sciences and coded as a separate category.[18] The vast majority of graduates had one degree only. However, a small proportion had multiple degrees and of these a small number of people were recoded as having a degree in clinical medicine based on later qualifications. All other graduates were coded by their first degree awarded prior to the 1991 Census. Degrees awarded after 1991 by subject were not considered as this question was not asked in subsequent censuses.

Work status variables were collected at the 2011 Census and used to adjust as a proxy for income as this is not collected in the Census. Respondents completed a tick box of options used to determine their participation in paid work the labour market in the week preceding each census. Working status in 2011 with those respondents considered to be ‘in work’ (this included working, on temporary sick leave, maternity leave, holiday or about to take up a job) with occupational social class based on the National Statistics Socio-economic classification (NS-SEC) with three categories was used as risk factor for analysis. The categories were: Managerial, administrative, and professional occupations; Intermediate occupations, Routine and manual occupations and never worked and unemployed combined.

For peer review only

Demographic and socioeconomic indicators in 2011 were included as potential covariates. Demographic variables included age and age-squared. The results are presented separately by sex.

Respondents were asked about self-rated health 'How is your health in general?' The outcome measure was good health and very good health combined compared to poor (fair, bad, and very bad health combined).

Patient and Public Involvement

This research was done without patient involvement. Patients were not invited to comment on the study design and were not consulted to develop patient relevant outcomes or interpret the results. Patients were not invited to contribute to the writing or editing of this document for readability or accuracy.

Ethics: the research project was approved by the ONS Longitudinal Study Research and Development Board. Project ID: 1007013

Results

Table 1 Sample characteristics

Cohort born before 1976 and survived until 2011 Census Completion who reported having a higher education degree in 1991 Census by NS-SEC and mean age

	1. Higher occupations	2. Intermediate occupations	3. Lower occupations /none	% 1. Higher occupations	Mean Age
Men					
D (Humanities)	573	960	251	32	62
A (Life Sciences)	701	408	115	57	61
B (Physical Sciences)	2853	1837	553	54	62
C (Social Sciences)	2212	1758	432	50	62
Clinical medicine	526	26	12	93	66
Missing degree subject	18	14	10	43	61
Women					
D (Humanities)	463	1877	347	17	60
A (Life Sciences)	478	540	88	43	58
B (Physical Sciences)	302	519	74	34	56
C (Social Sciences)	844	1882	261	28	57
Clinical medicine	278	26	10	89	62
Missing degree subject	suppressed	suppressed	suppressed	suppressed	54

Data Source ONS LS; analysis author's own

For peer review only

Table 2 Odds of having good or very good health in 2011 by degree status in 1991. Cohort born before 1976.

	Men	n=159,116			Women	n= 174,062		
	OR	p-value	Confidence	Interval	OR	p-value	Confidence	Interval
Does not have a degree in 1991	1.00				1.00			
Has a degree in 1991	1.92	<0.001	1.82	2.03	1.85	<0.001	1.73	1.98

Adjusted for Age, Age-squared and socio-economic status (NS-SEC); constant not shown

Data Source ONS LS; analysis author's own

Table 3 Odds of having good or very good health in 2011 by degree attained by 1991 Cohort born before 1976.

Subject grouped 2021 REF Panel classes (A-C) compared to Humanities (D)

		Men	n=13,390			Women	n= 8,143	
Subject	OR	p-value	Confidence	Interval	OR	p-value	Confidence	Interval
D (Humanities)	1.00				1.00			
A (Life Sciences)	1.16	0.163	0.94	1.44	0.95	0.663	0.77	1.18
B (Physical Sciences)	1.24	0.006	1.06	1.44	0.82	0.086	0.66	1.03
C (Social Sciences)	1.07	0.371	0.92	1.25	0.89	0.140	0.76	1.04
Clinical medicine	1.45	0.011	1.09	1.92	1.10	0.605	0.76	1.60
Missing degree subject	1.06	0.889	0.46	2.48	0.86	0.773	0.32	2.32

Adjusted for Age, Age-squared and socio-economic status (NS-SEC); constant not shown

Data Source ONS LS; analysis author's own

The majority of clinical medicine graduates (93% of men and 89% of women) were employed in higher occupational classifications. This is compared with 32% of male graduates in humanities subjects and 17% of female (Table 1). There were small differences between the mean age group of the groups of graduates analysed. The mean age of male and female clinical medicine graduates in the sample was higher than that of other male and female graduate groups respectively. The mean age of female graduates was lower than that of male by 2-7 years depending on degree subject (Table 1).

Male graduates had 92% higher odds of having good or very good health than male non-graduates after adjustment for age and socio-economic position (confidence intervals 1.82-2.03). Female graduates had 85% higher odds of having good or very good health than female non-graduates after adjustment for age and socio-economic position (confidence intervals 1.73-1.98) (Table 2).

Male clinical medicine graduates had 45% higher odds of having good or very good health than humanities graduates after adjustment for age and socio-economic position (confidence intervals 1.09.-1.92). Male physical sciences graduates also had higher odds of having good or very good health than

For peer review only

humanities graduates after adjustment for age and socio-economic position, but life sciences and social science graduates did not. There were no significant differences by degree subject for women (Table 3).

Conclusions: Male graduates in clinical medicine have higher odds of good or very good self-reported health. Knowledge of medicine may confer a health advantage for men above that of other degrees. The study of medicine may both inform personal health behaviour decisions and also lead to earlier self diagnosis through skills gained in research of clinical information and from knowing other experts in the medical field to consult. Additionally there are financial benefits of studying medicine that may explain the health advantage. Ross and Wu[19] found that fulfilling work and high income were very important in explaining the education-health link. As we found in previous work[8] medicine is one of the degree subjects which increases earnings the most - not only much more than the humanities and social science degrees, but also more than other sciences, and hence this could explain some of its strong positive impact of health. (This may also partly explain the association seen in physical sciences graduates).The census does not however collect details of income. The vast majority of clinical medicine graduates were employed in higher level occupations. Given the higher mean age of clinical medicine graduates if age selection were explaining the results we would explain this to reduce rather than increase the size of the association found suggesting this is not the explanation.

Why these benefits were only experienced by men might be explained by higher salaries of male clinicians or by the benefits of health-related knowledge mediating the gender differentials in health behaviours. There may also be selection bias, with men being more likely admitted to medical school and more likely to pursue a career in science generally than women, with men more likely to get employment and stay in a medical profession than women. Men's careers are less likely to be affected by family and childbearing responsibilities (though as this study looks at education rather than occupation the latter may be less of an issue). The mean age of the women in the sample was slightly younger than the men. There could also be effects on health where the educational qualification of the head of household may be more important, especially in households which are not headed by women. Cutler and Lleras-Muney[20] found that specific factual knowledge, e.g. on the harms of smoking and drinking, accounts for around 10% of the education gradient in health behaviours. We would obviously expect this specific factual knowledge to be highest for clinical medicine graduates. This could be further investigated by studying other graduates with health-related qualifications. The Medical Schools Selection Alliance details a minimum of three A levels (post-16) with qualifications usually in lab based sciences and often a third science subject for application to study medicine in the UK.[21] There are no post-16 academic subjects explicitly covering human health other than vocational and technical qualifications in Health and Social Care,[22] with Human Biology A level phased out in 2017.[23] Personal, social, health and economic education (PSHE) is a non-statutory subject on the English school curriculum in maintained schools and academies to age 16, though all state schools should make provision for its teaching.[24] Whether a compulsory GCSE and optional A level in a health related discipline would improve the population's health remains open for debate and to persuade medical schools whether this would form part of a suitable suite of qualifications with which to apply to medical school could also be challenging. This study has looked at graduates of medicine rather than those practising medicine. It is beyond the scope of the paper to look at how these outcomes may differ for those who study medicine but are employed in other fields though this is a potential area for future research, and gender may play an interesting role here. Also disentangling the effects of income might be considered if data on income were in future able to be linked to the ONS LS, perhaps as part of administrative based censuses.

For peer review only

a. Contributorship statement: NS and ODW devised the research idea and NS and ODW and WX collated the data and completed the analysis. LVDE and JB co-wrote the literature review and discussion with NS and ODW and provided comment on the full draft text.

b. Competing interests

None declared

c. Funding

CeLSIUS is funded by the Economic and Social Research Council Award Refs: ES/R00823X/1 ES/V003488/1

The work is the authors alone.

d. Data sharing statement

The data: Office for National Statistics Longitudinal Study (ONS LS) from which this panel is drawn are available from ONS via the Secure Research Service to approved researchers with approved projects.

Information and support for the LS for UK-based prospective and current users from the academic, statutory and voluntary sectors can be obtained from the Centre for Longitudinal Study Information and User Support (CeLSIUS) by emailing Celsius@ucl.ac.uk.

All other users should contact the ONS Longitudinal Study Development Team (LSDT): LongitudinalStudy@ons.gov.uk.

A step-by-step guide to using the LS is available from the CeLSIUS website www.ucl.ac.uk/celsius

The permission of the Office for National Statistics to use the Longitudinal Study is gratefully acknowledged, as is the help provided by staff of the Centre for Longitudinal Study Information & User Support (CeLSIUS). CeLSIUS is supported by the ESRC (Current Award Ref: ES/V003488/1).The authors alone are responsible for the interpretation of the data.

This work contains statistical data from ONS which is Crown Copyright. The use of the ONS statistical data in this work does not imply the endorsement of the ONS in relation to the interpretation or analysis of the statistical data. This work uses research datasets which may not exactly reproduce National Statistics aggregates.

1 Freedman V, Martin LG, "The role of education in explaining and forecasting trends in functional limitations among older Americans." Demography, 1999; **36**(4): 461-473

2 Elo IT, Preston SH. Educational differentials in mortality: United States, 1979-85. Soc Sci Med, 1996;**42**(1):47-57.

3 Valkonen T. Adult mortality and level of education: a comparison of six countries. In: John Fox, ed. Health Inequalities in European Countries. Aldershot: Gower Publishing Company Limited 1989: 142-162.

For peer review only

4 Walsemann KM, Bell BA, Hummer RA. Effects of Timing and Level of Degree Attained on Depressive Symptoms and Self-Rated Health at Midlife. Am J Public Health. 2012; **102**(3): 557–563.

5 Rogers RG , Everett BG, Zajacova A, Hummer RA. Educational Degrees and Adult Mortality Risk in the United States. Biodemography Soc Biol. 2010; 56(1): 80–99.

6 Kasey Buckles , Andreas Hagemann, Ofer Malamud Melinda Morrill Abigail Wozniak The effect of college education on mortality Journal of Health Economics 2016; **50**: 99–114

7 Bolton P. Education: Historical statistics Standard Note: SN/SG/4252 Social & General Statistics HoC Library, 2012.

8 Belfield C, Britton J, Buscha F, Dearden L, Dickson M, van der Erve L, Sibieta L, Vignoles A, Walker I, Zhu Y. The impact of undergraduate degrees on early-career earnings Institute for Fiscal Studies Department for Education 2018. https://assets.publishing.service.gov.uk/government/uploads/system/uploads/attachment_data/file/759278/The_impact_of_undergraduate_degrees_on_early-career_earnings.pdf

9 Chevalier A. Forschungsinstitut zur Zukunft der Arbeit Subject Choice and Earnings of UK Graduates IZA DP No. 5652 2011.

10 Heckman JJ, Humphries JE, Veramendi G, "Returns to Education: The Causal Effects of Education on Earnings, Health, and Smoking," Journal of Political Economy, University of Chicago Press, 2018; **126**(S1):197-246

11 Fletcher JM, Frisvold DE. Higher Education and Health Investments: Does More Schooling Affect Preventive Health Care Use? Journal of Human Capital 2009 **3**:2, 144-176

12 Belfield C, Britton J, van der Erve L, Shephard N. Where is the money going? Estimating the government cost of different university degrees IFS Briefing Note BN244 2019

13 Young H, Grundy E, O'Reilly D, Boyle P. Self-rated health and mortality in the UK: results from the first comparative analysis of the England and Wales, Scotland and Northern Ireland Longitudinal Studies Population Trends 2010;**139** 11-3514 ONS Graduates in the UK Labour Market: 2013 https://www.ons.gov.uk/employmentandlabourmarket/peopleinwork/employmentandemployeetypes/articles/graduatesintheuklabourmarket/2013-11-19

15 Shelton N, Marshall CE, Stuchbury R, Grundy E, Dennett A, Tomlinson J, Duke-Williams, Xun W (2019) Cohort Profile: the Office for National Statistics Longitudinal Study (The LS) International Journal of Epidemiology, 2019;**48**(2)383–384g,

16 Lynch K, Leib S, Warren J, Rogers N, Buxton J, Longitudinal Study 2001 – 2011 Completeness of census linkage Series LS No. 11 https://www.ons.gov.uk/file?uri=/aboutus/whatwedo/paidservices/longitudinalstudyls/longitudinalstudy20012011completenessofcensuslinkage.pdf (6 February 2018, date last accessed).

17 Office for National Statistics. Response Rates in the 2011 Census. Titchfield: Office for National Statistics, 2012. file:///C:/Users/stinks/AppData/Local/Temp/responseratesinthe2011censusv2dec2012final_tcm77-271600.pdf (6 February 2018, date last accessed).

For peer review only

19 UKRI Units of assessment 2019. https://www.ref.ac.uk/panels/units-of-assessment/ (6 February 2018, date last accessed).

19 Ross CE, Wu C-L. The Links Between Education and Health American Sociological Review 1995; **60** (5)719-745

20 Cutler and Adriana Lleras-Muney Understanding Differences in Health Behaviors by Education J Health Econ. 2010;**29**(1): 1–28.

21 Medical Schools Council Selection Alliance (2018) Entry requirements for UK medical schools 2018 entry https://www.medschools.ac.uk/media/2032/msc-entry-requirements-for-uk-medical-schools.pdf (6 February 2018, date last accessed).

22 UCAS (2018) UCAS Tariff tables Tariff points for entry to higher education from 2019 https://www.ucas.com/file/63536/download?token=lKi4qZse (6 February 2018, date last accessed).

23 Ofqual (2015) Further Decisions for Completing GCSE, AS and A Level Reform in 2017 https://assets.publishing.service.gov.uk/government/uploads/system/uploads/attachment_data/file/479635/2015-05-14-reform-of-gcses-as-and-a-levels-in-2017-may-2015.pdf (6 February 2018, date last accessed).

24 DfE (2020) Personal, social, health and economic (PSHE) education https://www.gov.uk/government/publications/personal-social-health-and-economic-education-pshe/personal-social-health-and-economic-pshe-education (25 November 2020, date last accessed).

For peer review only

STROBE Statement—checklist of items that should be included in reports of observational studies

	Item No.	Recommendation	Page No.
**Title and abstract**	1	(*a*) Indicate the study's design with a commonly used term in the title or the abstract	1
		(*b*) Provide in the abstract an informative and balanced summary of what was done and what was found	2
**Introduction**			
Background/rationale	2	Explain the scientific background and rationale for the investigation being reported	3-4
Objectives	3	State specific objectives, including any prespecified hypotheses	5
**Methods**			
Study design	4	Present key elements of study design early in the paper	5
Setting	5	Describe the setting, locations, and relevant dates, including periods of recruitment, exposure, follow-up, and data collection	5
Participants	6	(*a*) *Cohort study*—Give the eligibility criteria, and the sources and methods of selection of participants. Describe methods of follow-up<br>*Case-control study*—Give the eligibility criteria, and the sources and methods of case ascertainment and control selection. Give the rationale for the choice of cases and controls<br>*Cross-sectional study*—Give the eligibility criteria, and the sources and methods of selection of participants	5
		(*b*) *Cohort study*—For matched studies, give matching criteria and number of exposed and unexposed<br>*Case-control study*—For matched studies, give matching criteria and the number of controls per case	5
Variables	7	Clearly define all outcomes, exposures, predictors, potential confounders, and effect modifiers. Give diagnostic criteria, if applicable	5
Data sources/ measurement	8*	For each variable of interest, give sources of data and details of methods of assessment (measurement). Describe comparability of assessment methods if there is more than one group	5
Bias	9	Describe any efforts to address potential sources of bias	5
Study size	10	Explain how the study size was arrived at	5

Continued on next page

For peer review only - http://bmjopen.bmj.com/site/about/guidelines.xhtml

Quantitative variables	11	Explain how quantitative variables were handled in the analyses. If applicable, describe which groupings were chosen and why	5
Statistical methods	12	(*a*) Describe all statistical methods, including those used to control for confounding	5
		(*b*) Describe any methods used to examine subgroups and interactions	5
		(*c*) Explain how missing data were addressed	
		(*d*) *Cohort study*—If applicable, explain how loss to follow-up was addressed<br>*Case-control study*—If applicable, explain how matching of cases and controls was addressed<br>*Cross-sectional study*—If applicable, describe analytical methods taking account of sampling strategy	
		(*e*) Describe any sensitivity analyses	
**Results**			
Participants	13*	(a) Report numbers of individuals at each stage of study—eg numbers potentially eligible, examined for eligibility, confirmed eligible, included in the study, completing follow-up, and analysed	6
		(b) Give reasons for non-participation at each stage	
		(c) Consider use of a flow diagram	
Descriptive data	14*	(a) Give characteristics of study participants (eg demographic, clinical, social) and information on exposures and potential confounders	6
		(b) Indicate number of participants with missing data for each variable of interest	6
		(c) *Cohort study*—Summarise follow-up time (eg, average and total amount)	6
Outcome data	15*	*Cohort study*—Report numbers of outcome events or summary measures over time	*6*
		Case-control study—Report numbers in each exposure category, or summary measures of exposure	*6*
		Cross-sectional study—Report numbers of outcome events or summary measures	
Main results	16	(*a*) Give unadjusted estimates and, if applicable, confounder-adjusted estimates and their precision (eg, 95% confidence interval). Make clear which confounders were adjusted for and why they were included	6
		(*b*) Report category boundaries when continuous variables were categorized	
		(*c*) If relevant, consider translating estimates of relative risk into absolute risk for a meaningful time period	

Continued on next page

For peer review only

Other analyses	17	Report other analyses done—eg analyses of subgroups and interactions, and sensitivity analyses	
**Discussion**			
Key results	18	Summarise key results with reference to study objectives	7-8
Limitations	19	Discuss limitations of the study, taking into account sources of potential bias or imprecision. Discuss both direction and magnitude of any potential bias	7-8
Interpretation	20	Give a cautious overall interpretation of results considering objectives, limitations, multiplicity of analyses, results from similar studies, and other relevant evidence	7-8
Generalisability	21	Discuss the generalisability (external validity) of the study results	7-8
**Other information**			
Funding	22	Give the source of funding and the role of the funders for the present study and, if applicable, for the original study on which the present article is based	8

*Give information separately for cases and controls in case-control studies and, if applicable, for exposed and unexposed groups in cohort and cross-sectional studies.

Note: An Explanation and Elaboration article discusses each checklist item and gives methodological background and published examples of transparent reporting. The STROBE checklist is best used in conjunction with this article (freely available on the Web sites of PLoS Medicine at http://www.plosmedicine.org/, Annals of Internal Medicine at http://www.annals.org/, and Epidemiology at http://www.epidem.com/). Information on the STROBE Initiative is available at www.strobe-statement.org.

For peer review only - http://bmjopen.bmj.com/site/about/guidelines.xhtml
